# New Medium for Pharmaceutical Grade *Arthrospira*

**DOI:** 10.1155/2013/203432

**Published:** 2013-12-28

**Authors:** Amro A. Amara, Alexander Steinbüchel

**Affiliations:** ^1^Department of Protein Research, Genetic Engineering and Biotechnology Research Institute, (Mubarak) City for Scientific Research and Technology Applications, Universities and Research Centre District, New Borg El-Arab, P.O. Box 21934, Alexandria, Egypt; ^2^Institut für Molekulare Mikrobiologie ünd Biotekhnologie, Westfälishen Wilhelms-Universität Münster, Corrensstraße 3, 48149 Münster, Germany

## Abstract

The aim of this study is to produce a pharmaceutical grade single cell product of *Arthrospira* from a mixed culture. We have designed a medium derived from a combination between George's and Zarrouk's media. Our new medium has the ability to inhibit different forms of cyanobacterium and microalgae except the *Chlorella*. The medium and the cultivation conditions have been investigated to map the points where only *Arthrospira* could survive. For that, a mixed culture of pure *Chlorella* and *Arthrospira* (~90 : 10) has been used to develop the best medium composition that can lead to the enrichment of the *Arthrospira* growth and the inhibition of the *Chlorella* growth. To enable better control and to study its growth, an 80 l photobioreactor has been used. We have used high saline (2xA-St) medium which has been followed by *in fermentor* reducing its concentration to 1.5x. The investigation proves that *Chlorella* has completely disappeared. A method and a new saline medium have been established using a photobioreactor for *in fermentor* production of single cell *Arthrospira*. Such method enables the production of pure pharmaceutical grade *Arthrospira* for medicinal and pharmaceutical applications or as a single cell protein.

## 1. Introduction

Using algae as a food and medicine is deeply rooted in the human history. In ancient Egypt and today, farmers used to collect floating algae on the surface of the water to feed their domestic birds. In a harsh environment when the land resources food become rare, alkaline lakes play a significant role as an alternative source. The alkaline lakes enable the growth of one of the few nontoxic cyanobacterial species, *Arthrospira fusiformis *[1]. Humans learned early how to use the *Arthrospira *as a food source. Seeing the migrant birds feed safely on *Arthrospira*, such as lesser flamingoes (*Phoeniconaias minor *Geoffroy), encourages such use. Kebeda (1997) reported that in Ethiopia, farmers and herdsmen living in areas close to the soda lakes make their cattle drink *Arthrospira *water about once a month and believe that it has therapeutic effects and compensates for some lack in dietary food [2].

The invention of the microscope enabled Turpin in 1827 to identify and describe *Arthrospira *as spiral cyanobacteria [3]. Species of *Arthrospira *have been found in a variety of environments including soil, sand, marshes, brackish water, seawater, and freshwater [1, 4]. Rich (1931) has reported it as a dominant phytoplankton in a number of lakes in the Rift Valley of East Africa [5]. 113 years after its first microscopic identification, *Arthrospira *was reintroduced to the world by Dangeard (1940) from a sample collected by Mr. Cŕeach (a pharmacist) from a local market in Shad [1, 6]. *Arthrospira *contains high levels of proteins (50–70%), lipids (7–16%), vitamins, and omega-3 fatty acid [7–9]. For economic production of *Arthrospira*, it is usually cultivated in open ponds, so the absorbed solar energy is used to fix inorganic carbon. *Arthrospira *is produced in quantities exceeding 3000 tons/year of dry material [10]. In a survey concerning its production, Shimamatsu (2004) highlighted that its contamination by other algal species is one of the main problems concerning its production in the open ponds [10]. The analysis of its natural habitat enabled Zarrouk (1966) to introduce his famous alkaline medium [11]. Moreover, scientists have observed that it has become the most dominant species in high salt content lakes (>30 g/L) [12]. For better understanding and growth control, closed tubular photobioreactors with working volumes ranging from 5 to 36,000 l were used for the production of different cyanobacteria including *Arthrospira *[13–18]. *Arthrospira fusiformis *(formerly *Spirulina platensis*) is the main microalgae which is produced commercially in large scale as food or as nutraceutical food and single cell protein [19]. *Arthrospira *can grow in extremoalkalophilic and halophilic habitat as well as in fresh water [1]. It is used in the diets of fish and poultry and even sold as a healthy food [8]. It has been utilized for the production of cyanocobalamin (B12), antioxidant pigments like *β*-carotene, tocopherols, and *γ*-linolenic acid [7, 8, 20, 21].

The deep blue color of phycocyanin and other extractable pigments including myxoxanthophyll and zeaxanthin have been widely used as naturally occurring colorants for food additive purposes [22–25]. Phycocyanin and the *Arthrospira*'s exopolysaccharide have anticancer, antioxidant, antiviral, and anti-inflammatory activities and can be used as a tonic agent for the immune system [26, 27]. Producers used to increase alkalinity to reduce the number of algal species [10]. *Arthrospira *uses sunlight and CO_2_ to grow (autotrophic) or organic compounds (auxotrophic) or both (autoauxotrophic). For economic production of *Arthrospira*, alkaline open ponds are usually used for large-scale production. However, the major problem in its biomass production is the contamination with other algae and cyanobacteria which reduce the final product quality [10]. Few studies reported that *Arthrospira *can resist high salinity and that it becomes dominant in part of the years in its natural habitat [1, 2, 12]. While alkaline open ponds mimic the *Arthrospira *natural habitat, we are suggesting that salinity could play a significant role in *Arthrospira *enrichment and that the rain/evaporation cycle on the lake and the lake's surrounding area lead to salt accumulation, which leads to enrichment of *Arthrospira *over other algal and cyanobacterial species. Salt stress has been studied with less attention in other *Arthrospira*-related subjects [2, 28, 29].

In this study and for the first time, we introduce a simple strategy for single cell *Arthrospira *production in a photobioreactor using a new medium extracted from the nature and from the information in the literatures. We introduce two steps using the photobioreactor to eradicate *Chlorella *and to gain 100% pure *Arthrospira*.

## 2. Material and Methods

### 2.1. Chemical

All chemicals used were analytical grade and obtained from Sigma-Aldrich and Roth.

### 2.2. Cyanobacteria Strains

The extremoalkalophilic cyanobacteria strains used in this study were isolated from Lake Maryut, Alexandria, Egypt, and were identified using a light microscope as *Arthrospira* and *Chorella*. *Arthrospira* was identified by Sharaf et al. (2010) as *A. fusiformis* by sequencing and analysis of the PC-IGS regions in the gene of phycocyanin [26].

The *A. fusiformis* and *Chlorella* spp. strains were grown and cultivated routinely in Zarrouk's or George's media at room temperature (20–25°C) in lab condition under lamp/sunlight [11].

### 2.3. Media

#### 2.3.1. George's Medium

One liter of George's medium consists of peptone 1.00 g, KNO_3_ 0.20 g, K_2_HPO_4_ 0.02 g, MgSO_4_·7H_2_O 0.02 g, and Ferric citrate 0.035 g [30].

#### 2.3.2. Zarrouk's Medium [11]

One liter of Zarrouk's medium consists of (part A) NaHCO_3_ 16.80 g and K_2_HPO_4_ 0.50 g; (part B) NaNO_3_ 2.50 g, K_2_SO_4_ 1.00 g, NaCl 1.00 g, MgSO_4_·7H_2_O 0.20 g, EDTA-Na_2_·2H_2_O 0.08 g, CaCl_2_·2H_2_O 0.04 g, and FeSO_4_·2H_2_O 0.01 g; trace elements mixture A (part C 10 mL/l): 1.00 mL, trace elements mixture B (part D 1.0 mL/l): 1.00 mL; part C mg/L: H_3_BO_3_ 2.86, MnCl_2_·4H_2_O 1.810 g, ZnSO_4_·7H_2_O 0.222 MoO_3_·0.015, and CuSO_4_·5H_2_O 0.074 (the used amount is 10 mL/l); part D mg/L: NH_4_VO_3_ 22.9, NiSO_4_·7H_2_O 47.8, NaWO_2_ 17.9, Ti_2_(SO_4_)_3_·6H_2_O, and Co(NO_3_)_2_·6H_2_O 4.4 (the amount used was 1.0 mL/l) [11].

#### 2.3.3. Amara and Steinbüchel (A-St) Medium 1x

One liter medium of A-St consists of (part A) NaHCO_3_ 9.214 g, NaCO_3_ 7.143 g, and K_2_HPO_4_ 0.5 g; (part B) NaNO_3_ 1.5 g, K_2_SO_4_ 0.571 g, NaCl 1 g, MgSO_4_·7H_2_O 0.2 g, CaCl_2_·2H_2_O 0.012 g, FeSO_4_·2H_2_O 0.01 g, and EDTA-Na_2_·2H_2_O 0.08 g; (part C) ferric citrate 0.018 g; (part D) peptone 0.1 g; yeast extract 0.01 g.

### 2.4. Photobioreactor

**Figure 1:**
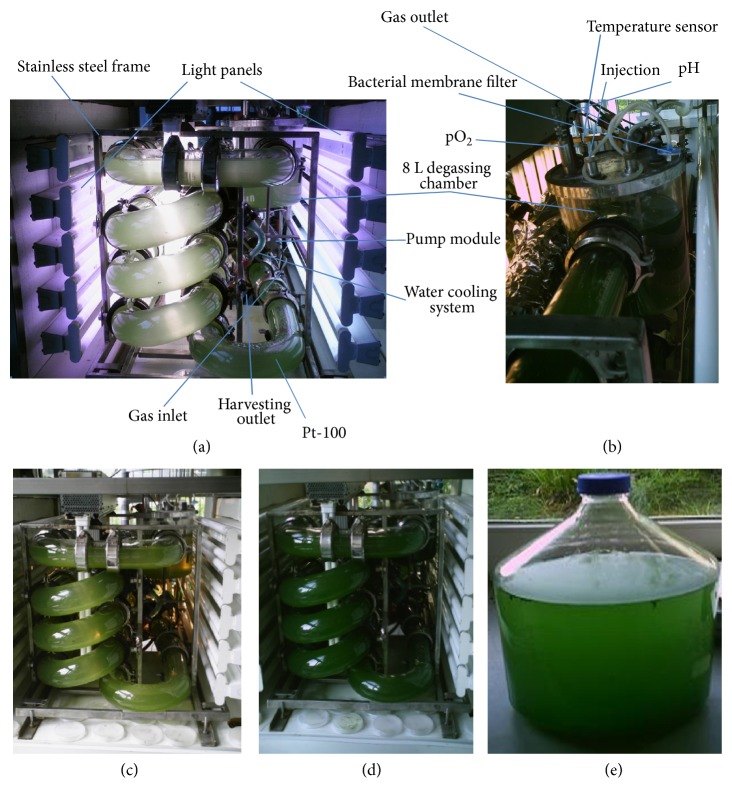
(a) Night profile of the photobioreactor showing the light system. (b) Degassing chamber and its stainless steel cover which contains different ports and growth mentoring probes (e.g., pH meter, CO_2_ and O_2_ electrodes, temperature sensor, and sample collection port). (c) Light growth of the *Arthrospira *after the medium dilution. (d) Heavy growth of the *Arthrospira *at the end of the cultivation process. (e) Static cultivation of *Arthrospira *using sun light only.

The photobioreactor used in this study was installed at the Institut für Molekulare Mikrobiologie ünd Biotekhnologie, Westfälishen Wilhelms-Universität, Münster, Germany, and has been previously described in detail by Hai et al. (2000) [16]. Its major features are as follows: it is made from helical Boresist DN80 glass tube (14m) (Schott Glaswerke, Mainz, Germany) and is connected to a degassing chamber (8.0 l) (Figures 1(a) and 1(b)). The photobioreactor surface/volume (s/v) ratio is about 44/m (Figure 1). The top of the degassing chamber was closed with a stainless steel plate providing ports (Figure 1(b)). A sealed pump module connected to a two-blade propeller was installed in the bottom of the photobioreactor and supplied with Pt-100 temperature sensor and contained an outlet to release the cell for harvesting (Figures 1(a), 1(b)). Three supplied light panels were placed in the interspaces and on both longitudinal sides of the photobioreactor. Each light panel contained 10 Osram Nature Deluxe, U- or L- shaped tubes (Osram, Munich, Germany) for maximum photon flux. The light intensity of each panel could be varied by dimming (Figures 1(a), 1(b), and 1(c)). Lighting with photon flux of approximately 0, 100, 400, and 600 *μ*E/m^2^ xs was applied to the cultivation process. Sterility of gas inlet or outlet was maintained by the ceramic bacterial filter.

### 2.5. In Flask Cultivation

250 mL flasks each contains 50 mL of Zarrouk's medium or George's medium were used for routine cultivation. When we used Zarrouk's medium, part A was autoclaved, while part B and the trace elements solutions A and B were sterilized separately using a bacterial membrane filter (0.22 *μ*m). For routine cultivation, light from a florescent lamp/sunlight and temperature ranging from 20 to 25°C under static condition were used.

### 2.6. In 20 l Bottle Cultivation

20 l bottle was used to draw the 2x medium from the photobioreactor during the dilution process. The removed 2x medium was adjusted to 1.5x and the *Arthrospira* was allowed to grow (under static condition same as above) (Figure 1(e)).

### 2.7. In Photobioreactor Medium Sterilization and Cultivation

The entire photobioreactor, except light panels and motor, was sterilized after adding 69 l of tap water containing part A from 2x A-St medium using model 6612-1 ED chamber autoclave (KSG Sterilization GmBH, Olching, Germany). Part B and part C were sterilized separately using sterile bacterial membrane filter (0.22 *μ*m). The total volume of the medium constituents has been made to 1 l and added separately to the photobioreactor to achieve a total volume of 70 l. Part 4 which contains the yeast extract/peptone was autoclaved using normal autoclave. The electrodes and the ceramic filters were sterilized separately.

### 2.8. Cultivation Condition

The photobioreactor enabled automatic control. The temperature was set to 30°C and was adjusted automatically. The stirrer speed (motor speed), the pO_2_, and the pH were monitored automatically. The parameters at the starting point were pH 9.34, pO_2_ 80, motor speed 0 rpm, temperature 26°C, and 600 *μ*E/m^2^ xs. Samples were taken regularly and whenever possible.

### 2.9. Cell Dry Weight (CDW)

CDW was calculated by correlating weight to volume as g/L. Filter papers or gauze, or both, were used. The paper or gauze was weighed before being used and dried after filtration, and the weight correlated to the volume was used as CDW g/L.

### 2.10. Microscopic Examination

Samples were drawn from the photobioreactor regularly and whenever possible and were examined using light microscope.

### 2.11. Salinity and Media Composition Study

A comparison study was done between the different used media to understand their different effect on *Arthrospira *growth based on their cation, anion, and salt constituents.

### 2.12. Arthrospira Total Biomass Collection

For collecting the total biomass of the *Arthrospira*, filtration was performed using sponge covered with gauze.

### 2.13. Biomass Drying

The *Arthrospira *wet biomass was dried under aseptic conditions by passing sterile air over the *Arthrospira *biomass in a sterile microbial cultivation cabinet.

## 3. Results and Discussions

**Figure 2:**
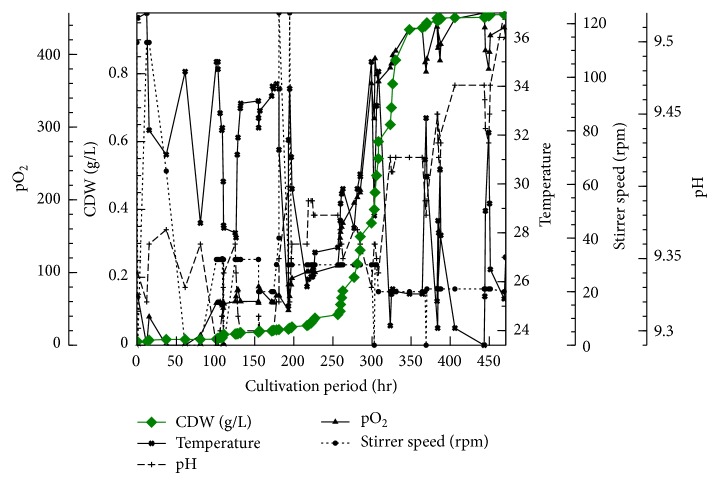
The overall cultivation process using photobioreactor. The heavy green line shows the CDW g/L.

During our testing of media compositions using *Chlorella* and *Arthrospira* (both isolated by methods other than media enrichment) in one flask alone or plus a mixture of other unidentified algae (data not shown), we observed that a high amount of Na_2_CO_3_ (2% w/v) enables *Arthrospira *and *Chlorella* to flourish but inhibits growth of other algae (Tables 1–3). Given that *Arthrospira *can grow under auxotrophic condition, we used the peptone/yeast extract and tap water instead of the trace elements mixture. We have selected the components of our new medium from both George's and Zarrouk's media. George's medium is a common medium for algae cultivation. George's medium contains peptone and the tap water in its gradients. The constituents which are used for Zarrouk's medium have been selected based on being a selective medium for the *Arthrospira*. By accident, we used a double concentration (2x) of A-St medium. The 2x medium upon its used found to be able to cure all the *Chlorella*. For that we developed a two-step strategy: first using 2x medium to eradicate *Chlorella* and then diluting the 2x medium to 1.5x so that *Arthrospira *can flourish again (Tables 1–3). We used a light microscope to evaluate the process and showed that after 2 days of cultivation *Chlorella* was completely eradicated. Moreover, *Arthrospira* survived in 2x of our medium without the yeast extract/peptone mixture. This initiated the idea to establish an *in fermentor* purification for *Arthrospira* heavily contaminated by *Chlorella* (10 *Arthrospira* : 90 *Chlorella*) (Figures 1(a), 1(b), 1(c), 1(d), and 1(e)). The cell dry weight (CDW) for *Arthrospira* in A-St 2x and 1.5x media is plotted on a linear or an exponential scale in Figures 1 and 2. The other parameters have been plotted against time in Figures 2 and 6.

**Figure 3:**
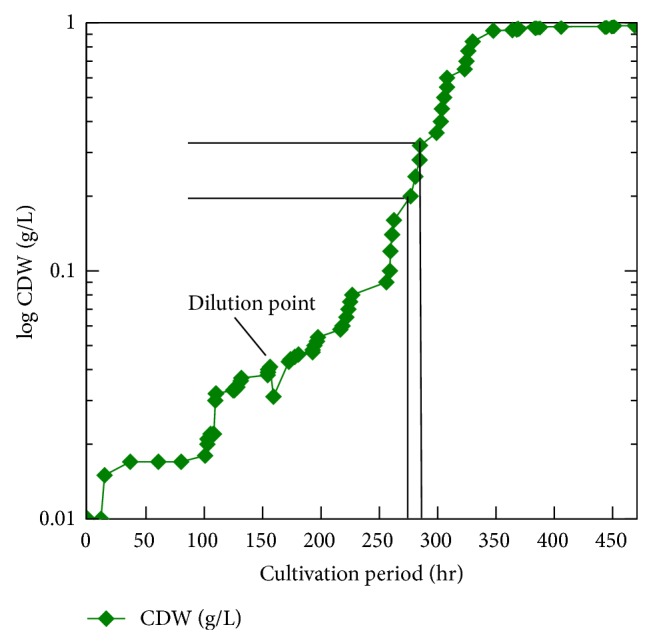
Log CDW for the *Arthrospira *overall cultivation process (A-St 2x and 1.5x media).

**Figure 4:**
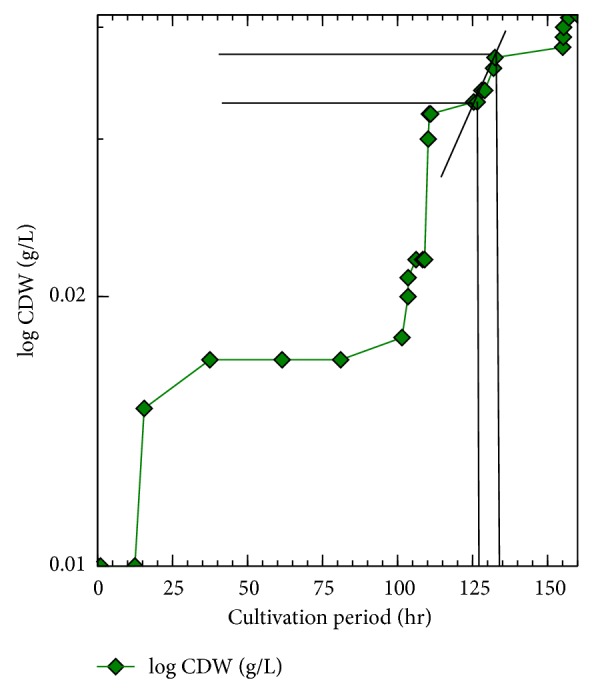
Log CDW for the *Arthrospira* at A-St 2x medium.

The growth curve of *Arthrospira* represents a typical growth curve of most microbes. It shows a typical lag, log, and stationary phase (Figure 2). The total yield of *Arthrospira* biomass as CDW was 0.979 g/L (68.53 g/70 l). As shown in Figure 2, the growth of *Arthrospira* in 2x medium is very weak even though it is still similar to the microbial growth curve in Figures 2, 3, and 4. The calculated productivity *P* = (*x*_end_ − *x*_start_)/*t*_end_ − *t*_start_, where *x*_end_ and *x*_start_ are the CDW g/L at the 0 and end points and *t*_end_ and *t*_start_ are the times at 0 and end points. For cultivation using the 2*x* medium, the *P*_1_ = 0.00013 g/L/h, the calculated specific growth rate *μ* = (ln *x*_2_ − ln *x*_1_)/(*t*_2_ − *t*_1_)*μ* = 0.0215/h, and the calculated generation time *g* = ln2/*μ*, *g* = 32.23 h. By diluting the medium concentration to 1.5x as in Figures 3 and 4, the above values have been increased significantly, where *P*_2_ = 0.00304 g/L/h, *μ* = 0.0406 g/h, and *g* = 17.068. The significant increase in the *P*_increase_ and *μ* and the decrease in *g* at 1.5x medium prove that salinity is an essential factor in the *Arthrospira *enrichment. The increase in each of *the P*, *μ*, and *g* has been calculated from the following formula:

(1) %Xincrease=[(X2∗100)X1]−100,

where *X* is either *P* or *μ* or *g*.

The calculated *P*_increase_ = 2238.4%, *μ*_increase_ = 88.83%, and *g*_increase_ = −47.04%.

*μ* and *g*, for each of 2x and 1.5x have been calculated at nearly the same time from each starting point. We selected the best fitted points in the exponential growth curve at 2x medium cultivation as in Figure 4 from the following equation: for 2x cultivation the starting point (0 point) is at 0 h and the end point is at 160 h, while for 1.5x cultivation the 0 point is at 160 h and the end point is at 471 h.

The best representative points in both cultivations have been calculated from the following formula:

(2) Point2=(Point1−Point0(1))∗2+(Point0(2)−Point1)  Point2=(126−0)∗2+(160−126)=286 h.

Both of the 2x and 1.5x slopes have been selected from the best 4 matching points as in Figures 3 and 4, where Point_0(1)_ and Point_0(2)_ are the starting points for each cultivation, respectively.

Analysis of the five media used is summarized in Table 1, and their constituents of anion, cation, and Na salt were calculated as Mol/l. A-St medium at 1x concentration is similar to Zarrouk's medium, while 2x medium contains a high amount of salt. A-St 1.5 medium is located in the middle between x2 and both of A-St x1 and Zarrouk's medium, and it gave the best result. The hardness of each medium has been calculated from the following formula:

(3) M CaCO3M Ca=100.140.1=2.5M CaCO3M Mg=100.124.3=4.1[CaCO3]=2.5[Ca+2]+4.1  [Mg2+].

**Figure 5:**
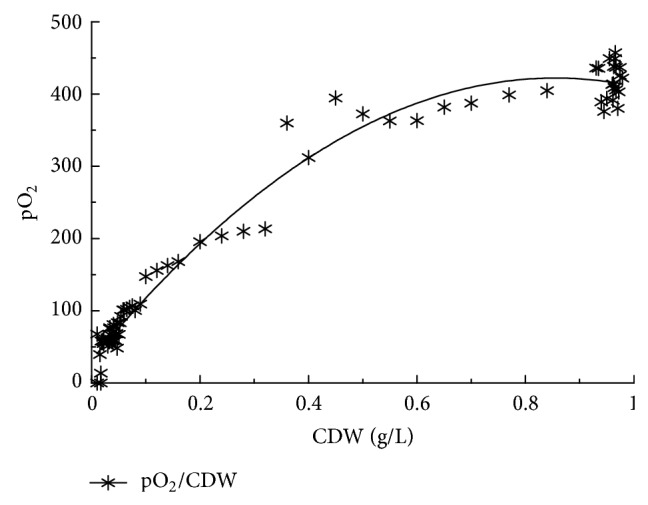
Fitted curve of the CDW against pO_2_.

**Figure 6:**
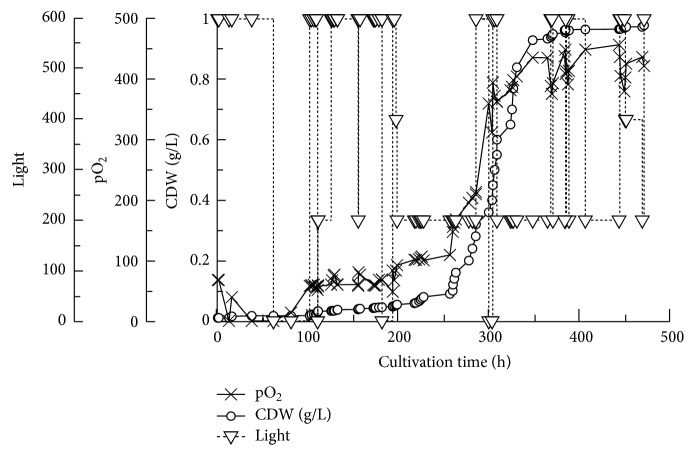
The effect of the light in both pO_2_ and CDW g/L.

The different media used, except George's medium, are very hard (Table 2). The increase in the Ca and Mg ions leads to an increase in the media total hardness. There is a reverse relationship between the % of *Chlorella *and *Arthrospira *in different Na salt concentrations where *Chlorella *decreases with increasing new salt concentration and *vice versa *in the case of *Arthrospira *(Table 3). The relationship between pO_2_ and the *Arthrospira *CDW is linear as shown in Figures 2 and 5; that is, an increase in the *Arthrospira *CDW causes an increase in pO_2_. Different light intensities, including 0, 200, 400, and 600, have been used as shown in Figure 6, and the pO_2_ is more sensitive for monitoring the effect of light in the cultivation process. In the lag and log phase, the change in light intensity is not clearly detected except at a few points (e.g., at 300 h). This is because in the lag phase cell growth and division are very slow and in the log phase they are very high. The change could not be detected clearly in both growth phases; however, in the stationary phase, where the growth becomes more stable and constant, the effect has been clearly observed and showed that the decrease in light intensity led to a decrease in growth as well as in the amount of the released pO_2_ as in Figure 6.

*Arthrospira* can exist in harsh conditions, and it can even be used in bioremediation of toxic elements such as lead and other toxic elements and compounds [31, 32]. Heterotrophic metabolism is faster than autotrophic [33]. Aiming to eradicate *Chlorella*, we used stresses which included high levels of alkalinity, salinity, and autotrophic growth. *Arthrospira* proved to be able to survive in these conditions while *Chlorella* did not. In the photobioreactor, we used autotrophic conditions first to put *Arthrospira* and *Chlorella* in maximum expected stress (we added yeast extract/peptone mixture after diluting the medium to 1.5x). Temperature, which can affect pO_2_ and pH, was set to 30°C. After 160 h of these harsh conditions, the 2x A-St medium was then diluted to 1.5x, and the *Arthrospira* growth started to show an increase in its biomass as shown in Figures 1(b) and 1(c).

We stopped the process after about 471 h after the growth rate had become saturated. The increase in the biomass is relatively equal to the increase in the pO_2_ (% of saturation) which is logical, while the elevation of O_2_ is an indicator of the cell growth and multiplication. pO_2_ and pH are negatively affected by temperature. The mixing process leads to an increase in the fermentation process temperature. However, the range of the change in each of the pH and O_2_ is narrow. The sudden change in pO_2_ amount means that there is a direct effect on the *Arthrospira* growth as in Figures 2, 3, and 4. The *μ*, *P*, and *g* parameters of the growth before and after the medium dilution prove that 1.5x A-St medium is better for growing *Arthrospira* than 2x A-St medium. This is another proof about the role of salinity level in the growth of cyanobacteria. Therefore, the analysis of the different media which have been used according to their anion, cation, and salinity is shown in Table 1. According to the data in Table 3 as well as that in Tables 1 and 2, salinity is the major factor which leads to the eradication of the *Chlorella*. On the other hand, *Arthrospira* proves to be a powerful strain that could resist different kind of stresses, especially those used in our study.

This study did not investigate conditions for *Arthrospira* overproduction or its active constituents' analysis, which should be covered in future studies. However, it clearly proves the role of salinity in its growth, in the enrichment, and in the inhibition of other algal species. Other *Arthrospira* should be tested as case-by-case study because there is great variability in the genotype and the stage of the growth cycle (as well as many other factors) as reported by Ruengjitchatchawalya et al. (2002) [34]. By mimicking its natural habitat and using the photobioreactor within* fermentor* partial randomization for its cultivation conditions, we gain a better understanding for *Arthrospira* growth conditions.

## 4. Conclusion

In conclusion, salinity was the major factor which leads to the dominance of *Arthrospira *rather than the alkalinity. Purifying *Arthrospira* from contaminated algae to reach a pharmaceutical grade using 2x/1.5x A-St media is a step to improve the *Arthrospira* quality. Tap water and yeast extract/peptone mixture can substitute the trace elements in the *Arthrospira* commercial production. The temperature could change the amount of pO_2_ and the pH value either by direct effect or by inducing chemical or physical changes. *Arthrospira* can sense any change in its environment. *Arthrospira* has proved to be more environmentally adapted to stress than *Chlorella*. In this study, we succeeded in developing a medium and cultivation conditions enabling the enrichment of the *Arthrospira* and the inhibition of the other algal species based on salinity. Using the photobioreactor and conducting a complete cultivation process (lag-log-stationary phases) have been proved to be the most efficient and quickest ways to understand the different responses of *Arthrospira* to the different modifications during its cultivation conditions. This study will open the way to produce a pure culture of *Arthrospira*. Most methods used nowadays do not guarantee pure *Arthrospira* production which affects its product quality. Our new method is not expensive, reliable, and cost effective. The medium which was taken from the fermentor during the dilution step can be diluted and reused. *In fermentor Arthrospira* can be produced without any algal or cyanobacteria contaminant. This will enable the production of pure *Arthrospira* and enables the purification of a previously produced contaminated *Arthrospira* (e.g., obtained from open ponds).

## Figures and Tables

**Table 1:** Different used media and their compositions (salt, cation, and anion).

Compound	Cation	Anion	Cation subtotal mass (g/mol)	Anion subtotal mass (g/mol)	2x Amara-Steinbüchel				1.5x Amara-Steinbüchel				1x Amara-Steinbüchel				George's medium				Zarrouk medium			
					2x 1 L	Cation Mol	Anion Mol	Na Mol	1.5x 1 L	Cation Mol	Anion Mol	Na Mol	1x 1 L	Cation Mol	Anion Mol	Na Mol	1x 1 L	Cation Mol	Anion Mol	Na Mol	1x 1 L	Cation Mol	Anion Mol	Na Mol
NaHCO_3_	Na	HCO_3_	22.99	61.02	18.43	0.8015	0.3020	0.8015	13.8214	0.6011	0.2265	0.6011	9.2142	0.4007	0.1510	0.4007					16.8000	0.73075	0.2753	0.7307
NaCO_3_	Na	CO_3_	22.99	60.01	14.29	0.6213	0.2380	0.6213	10.7142857	0.4660	0.1785	0.4660	7.1428	0.3106	0.1190	0.3106								
K_2_HPO_4_	K	HPO_4_	78.2	95.98	1.00	0.0127	0.0104		0.7500	0.0095	0.0078		0.5000	0.0063	0.0052		0.0200	0.0002	0.0002		0.5000	0.0063	0.0052	
NaNO_3_	Na	NO_3_	22.99	62.01	3.00	0.1304	0.0483	0.1304	2.2500	0.0978	0.0362	0.0978	1.5000	0.0652	0.0241	0.0652					2.5000	0.1087	0.0403	0.1087
K_2_SO_4_	K	SO_4_	96.07	78.2	1.14	0.0118	0.0146		0.8571	0.0089	0.0109		0.5714	0.0059	0.0073						1.0000	0.0104	0.0127	
NaCl	Na	Cl	22.99	35.45	2.00	0.0869	0.0564	0.0869	1.5000	0.0652	0.0423	0.0652	1.0000	0.0434	0.0282	0.04349					1.0000	0.0434	0.0282	0.0434
MgSO_4_	Mg	SO_4_	24.31	96.07	0.31	0.0125	0.0031		0.2295	0.0094	0.0023		0.1530	0.0062	0.0015		0.0200	0.0008	0.0002		0.2000	0.0082	0.0020	
CaCl_2_	Ca	Cl	40.08	70.91	0.02	0.0003	0.0002		0.01125	0.0002	0.0001		0.0075	0.0001	0.0001						0.0400	0.0009	0.0005	
FeSO_4_	Fe	SO_4_	55.85	96.07	0.02	0.0002	0.0001		0.01215	0.0002175	0.0001		0.0081	0.0001	0.00008						0.0100	0.0001	0.0001	
EDTA Na_2_	Na	EDTA	45.98	290.23	0.14	0.0031	0.0004	0.0034	0.1083	0.0023	0.0003	0.0026	0.0722	0.0015	0.0002	0.0017					0.0800	0.0017	0.0002	0.0017
Ferric Citrate	Ferric	Citrate	55.85	189.1	0.04	0.0006	0.0001		0.0262	0.0004	0.0001		0.0175	0.0003	0.00009		0.0350	0.0006	0.0001					
KNO_3_	K	NO_3_	39.1	62.01													0.2000	0.0051	0.0032					

Total			583.25	1293.13	40.37	1.6821	0.6741	1.6439	30.2803	1.2616	0.5056	1.2329	20.1868	0.8410	0.3370	0.8219	0.2750	0.0068	0.0038	0.0000	22.1300	0.9109	0.3648	0.8847

**Table 2:** Water hardness of the different used media.

	A-St 2x	A-St 1.5x	A-St 1x	George's	Zarrouk's
MgSO_4_	0.0125	0.0094	0.0062	0.0008	0.0082
CaCl_2_	0.0003	0.0002	00001	—	0.0009
Equivalent mg L^−1^ or ppm of CaCO_3_	1.28*e* ^+3^	957	628	—	907
French degree	128	95.7	62.8	—	90.7
German degree	71.4	53.6	35.2	—	50.8
English degree	89.3	67.0	43.9	—	63.8

Water hardness	Very hard water	Very hard water	Very hard water		Very hard water

**Table 3:** The % of the cells for both *Chlorella* and *Arthrospira* in different media.

Media names	Na	*Chlorella *	*Arthrospira *
George's	0	99	1
A-St 1x	0.8129	92	8
Zarrouk's	0.8847	90	10
A-St 1.5x	1.2329	30	70
A-St 2x	1.6439	0	100
